# Bacterial adaptation during chronic infection revealed by independent component analysis of transcriptomic data

**DOI:** 10.1186/1471-2180-11-184

**Published:** 2011-08-18

**Authors:** Lei Yang, Martin Holm Rau, Liang Yang, Niels Høiby, Søren Molin, Lars Jelsbak

**Affiliations:** 1 Department of Systems Biology, Technical University of Denmark, DK-2800, Lyngby, Denmark; 2 Faculty Of Health Sciences, Department of International Health, Immunology and Microbiology, University of Copenhagen, DK-2200, København N, Denmark

## Abstract

**Background:**

Bacteria employ a variety of adaptation strategies during the course of chronic infections. Understanding bacterial adaptation can facilitate the identification of novel drug targets for better treatment of infectious diseases. Transcriptome profiling is a comprehensive and high-throughput approach for characterization of bacterial clinical isolates from infections. However, exploitation of the complex, noisy and high-dimensional transcriptomic dataset is difficult and often hindered by low statistical power.

**Results:**

In this study, we have applied two kinds of unsupervised analysis methods, principle component analysis (PCA) and independent component analysis (ICA), to extract and characterize the most informative features from transcriptomic dataset generated from cystic fibrosis (CF) *Pseudomonas aeruginosa *isolates. ICA was shown to be able to efficiently extract biological meaningful features from the transcriptomic dataset and improve clustering patterns of CF isolates. Decomposition of the transcriptomic dataset by ICA also facilitates gene identification and gene ontology enrichment.

**Conclusions:**

Our results show that *P. aeruginosa *employs multiple patient-specific adaption strategies during the early stage infections while certain essential adaptations are evolved in parallel during the chronic infections.

## Background

Bacterial infections are one of the major causes of mortality among human and animals in the world [1]. Understanding adaptation of bacterial pathogens to the dynamic and hostile environment is crucial for improvement of therapies of infectious diseases. Bacteria associated with chronic infections in patients suffering from e.g. AIDS, burn wound sepsis, diabetes and cystic fibrosis (CF) are ideal objects for studying bacterial adaptation.

In airways of CF patients, mucus forms a stationary and thickened gel adhering to the epithelial lining fluid of the airway surfaces, which affects the mucociliary escalator and results in impaired clearance of inhaled microbes [2]. CF patients suffer from chronic and recurrent respiratory tract infections which eventually lead to lung failure followed by death. *Pseudomonas aeruginosa *is one of the major pathogens for CF patients and is the principal cause of mortality and morbidity in CF patients [3]. Early *P. aeruginosa *infection in CF patients is characterized by a diverse of *P. aeruginosa *strains which have similar phenotypes as those of environmental isolates [4,5]. In contrast, adapted dominant epidemic strains are often identified from patients chronically infected with *P. aeruginosa *from different CF centers [4,6,7]. Once it gets adapted, *P. aeruginosa *can persist for several decades in the respiratory tracts of CF patients, overcoming host defense mechanisms as well as intensive antibiotic therapies [8].

As *P. aeruginosa *has been sequenced, transcriptome profiling (e.g. microarray analysis and RNA-Seq) becomes a convenient approach for characterizing biological differences among different *P. aeruginosa *clinical isolates from CF patients. Transcriptome profiling enables researchers to measure genome-wide gene expressions in a high-throughput manner thus can provide valuable information for *P. aeruginosa *adaptation during infections. However, the interpretation of transcriptomic data is a great challenge for researchers due to the complexity and noise. Clinical strains isolated from different patients have adapted to distinct host environments since patients vary in their ages, infection histories and medical treatments (e.g. different kinds of antibiotics and their dosages). Therefore, researchers need to reduce dimensionality and extract the underlying features from the multi-variable transcriptomic dataset.

Principle component analysis (PCA) is a classic projection method which is widely used to accomplish the above mentioned tasks [9]. PCA transforms a number of correlated variables into a smaller number of uncorrelated variables called principal components (PC). The first PC captures as much of the variability in the data as possible, and each succeeding PCs capture as much of the remaining variability as possible. However, the constraint of mutual orthogonality of components implied in classical PCA methods may not be appropriate for the biological systems. Recently, independent component analysis (ICA), which decomposes input data into statistically independent components, was shown to be able to classify gene expressions into biologically meaningful groups and relate them to specific biological processes [10]. ICA has been successfully applied by different research groups to analyze transcriptomic data from yeast, cancer, Alzheimer samples and is shown to be more powerful at feature extraction than PCA and other traditional methods for microarray data analysis [11-13]. In a study by Zhang *et al*., ICA was used to extract specific gene expression patterns of normal and tumor tissues, which can serve as biomarkers for molecular diagnosis of human cancer type [14]. Yet to the best of our knowledge, there have been no reports of application of ICA to the study of bacterial transcriptomic data from chronic infections.

In this study, we applied ICA to project the transcriptomic data of 26 CF *P. aeruginosa *isolates into independent components. *P. aeruginosa *genes are unsupervisedly clustered into non-mutually exclusive groups. Each retrieved independent component is considered as a putative adaptation process, which is revealed by the functional annotations of genes that give heavy loadings to the component.

## Results

**Figure 1:**
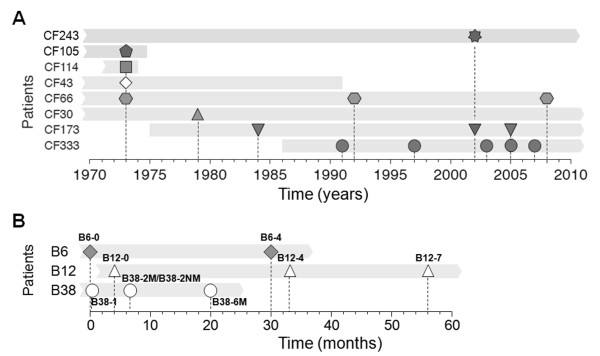
**Isolate sampling points and patient life span**. *P. aeruginosa *isolates were collected from eleven different CF patients during a 35-y time period. Bacterial isolates are represented by the different symbols and patient life span is represented gray bars. This figure is adapted from Yang et al., 2011 [8].

The *P. aeruginosa *microarray dataset is mainly generated from two studies (Figure 1). In the first study, *P. aeruginosa *strains were collected from a group of patients since 1973 (Figure 1A) [8]. Those isolates represent different *P. aeruginosa *clonal lineages adapted from early stage infection to chronic stage infection. In the second study, *P. aeruginosa *strains were collected from a group of CF children since 2006, except the B38-2NM is an isogenic non-mucoid strain of the mucoid B38-2M isolate generated *in vitro *by allelic replacement of its *mucA *allele (Figure 1B) [5]. Those isolates represent different *P. aeruginosa *clonal linages adapted in early stage infection at nowadays. As a control, a well studied wild-type *P. aeruginosa *laboratory strain PAO1 was included in the dataset. The microarray dataset was prepared as matrix ***X ***which contains ***n ***(26) samples and ***m ***(5900) columns. We modeled the whole gene expression in a cell as a mixture of independent biological process by using FastICA method [15]. The *P. aeruginosa *microarray data matrix ***X ***was decomposed by FastICA into latent variable matrix ***A ***(26 × 26) and gene signature matrix ***S ***(26 × 5900).

### ICA improved clustering patterns of *P. aeruginosa *microarray data

**Figure 2:**
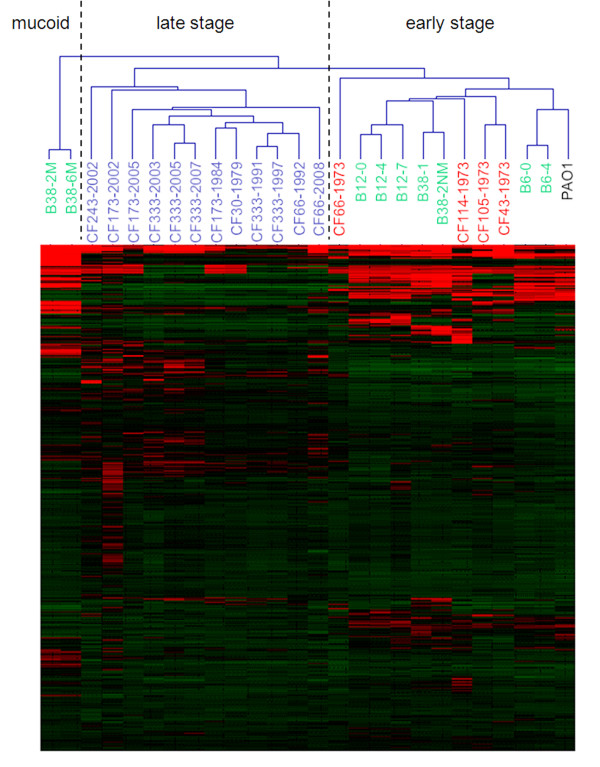
**Hierarchical clustering of the normalized raw data using Euclidean distances**. Red/green blocks represent signal increase/decrease respectively.

**Figure 3:**
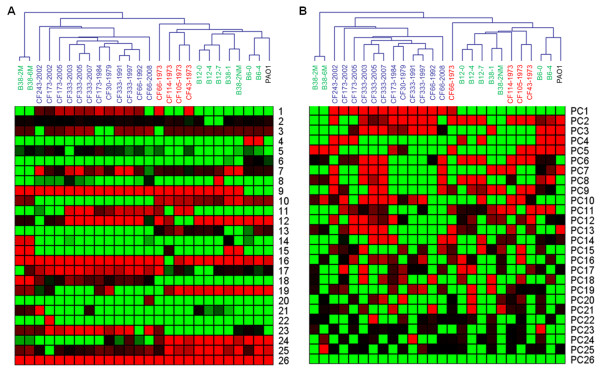
**Hierarchical clustering of the ICA and PCA outputs**. (A) Hierarchical clustering of the ICA outputs with the last 'common' components of matrix ***A ***removed. (B) Hierarchical clustering of the principle components, with the number of the principle components k = 26.

Unsupervised hierarchical clustering was applied to the original normalized data, the outputs of ICA (latent variables) and the outputs of PCA (principle components), respectively. For the original data, the *P. aeruginosa *isolates were grouped into three distinct groups: an early stage infection group, a late stage infection group and a mucoid strain group (Figure 2). The early stage infection isolates were grouped together with the PAO1 strain, which indicates that they have not gained extensive adaptations. However, the clustering did not fully discriminate the early stage isolates (CF114-1973, CF105-1973 and CF43-1073, strain names marked in red color) of Yang's study [8] from the early stage isolates (B12-0, B12-4, B12-7, B38-1, B38-2NM, B6-0 and B6-4, strain names marked in green color) from Rau's study [5]. In contrast, the clustering dendrogram from ICA outputs showed better separation of the early stage isolates from the two different studies (Figure 3A). The CF114-1973 was clustered together with the CF105-1973 and CF43-1973 from the ICA outputs (Figure 3A). This indicates that these two groups of early stage isolates have distinct physiology. Clustering dendrogram from PCA outputs (Figure 3B) generated the same pattern as the one generated from the original data (Figure 2). These results showed that ICA is better than PCA in filtering noisy and extracting important features from microarray data.

### ICA identified significant genes for adaptation of *P. aeruginosa *to the CF airways

**Figure 4:**
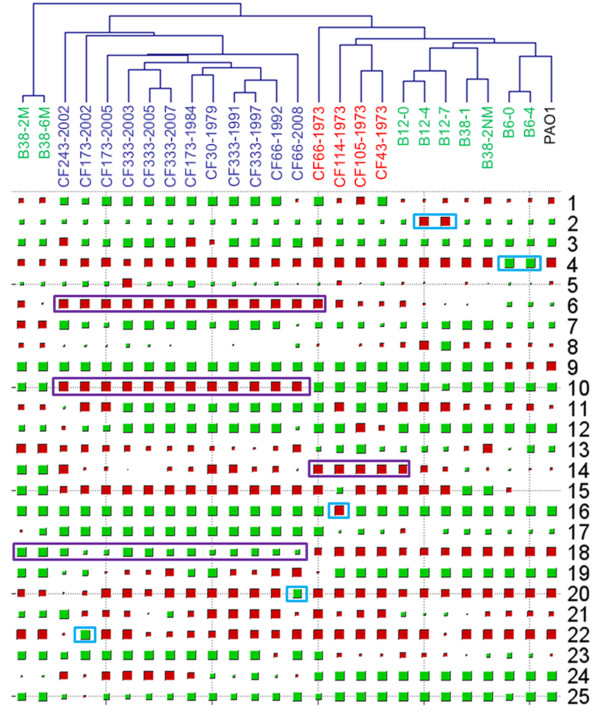
**Hinton diagram representation of latent variable matrix *A***. The size of each square corresponds to the amount *a_nm _*of component *m *in sample *n*. Red and green represent positive and negative values, respectively.

The ICA output matrix ***A ***contains the weight with which the expression levels of the ***m ***genes contribute to the corresponding observed expression profile. Thus, together with the clustering dendrogram, ICA derived components can also implicate individual genes that give the strongest contribution to that component. To achieve this purpose, we firstly used Hinton diagram to represent the matrix ***A ***derived by FastICA (Figure 4). As previously reported [13], the values of the last latent variable are similar across all samples and have no biological relevance. Thus the last latent variable was removed from matrix ***A ***before the Hinton diagram analysis. From this figure, we can identify the latent variables related to adaptation of different *P. aeruginosa *isolates (Table 1).

**Table 1:** Latent variables related to specific adaptation

Latent variables	Related strains	Functions of selected enriched genes by ICA	
		***Up regulated***	***Down regulated***

2	B12-4, B12-7	Antibiotic resistance Iron metabolism Citronellol/leucine catabolism	-

4	B6-0, B6-4	LPS modification	Flagellum biogenesis

16	CF114-1973	Fimbrial biogenesis	-

20	CF66-2008	LPS modification	-

22	CF173-2002	-	-

14	Early stage isolates from 1973	Type III secretion	-

6	Late stage isolates	Antimicrobial peptide tolerance	-

10	Late stage isolates	Potassium uptake system	Quorum sensing

18	Late stage isolates	Alginate biosynthesis	Motilities

**Figure 5:**
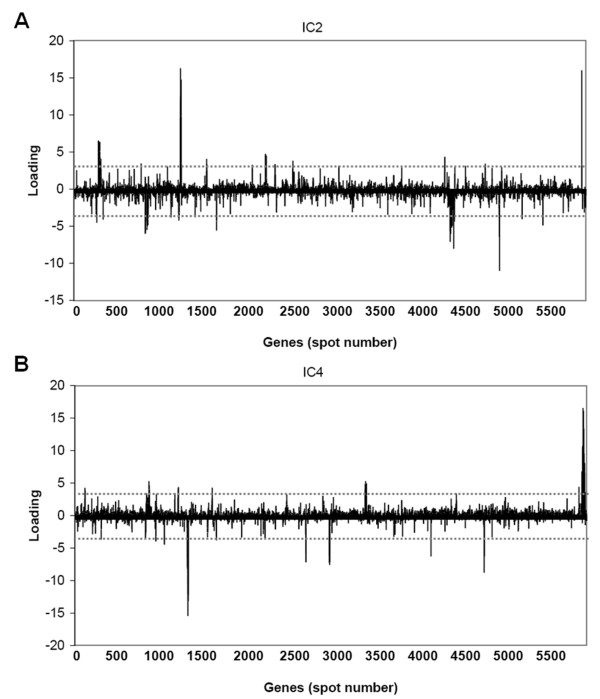
**The selected significant genes for 2-th (A) and 4-th (B) gene signatures**. Genes with loadings exceeding the chosen percentile lines were considered significant. Positive and negative loadings correspond to up-and down-regulation of expressions, respectively.

Afterwards the corresponding gene signatures (ICs) of the identified latent variables could be found through matrix ***S***. Figure 5 shows the corresponding gene signatures in matrix ***S ***(2-th and 4-th rows of ***S ***as example) for the 2-th and 4-th components in matrix ***A***. Depending on the loadings of latent variables, the genes with loading that exceed the chosen threshold (4 or 2) were selected as the most significant genes contributing to that component. Some of the highlighted significant genes identified through the selected latent variables are shown in Table 1. A full list of identified significant up- and down-regulated genes corresponding to the selected latent variables of Table 1 could be found in Additional file 1, Table S1.

ICA revealed common adaptations shared by a group of *P. aeruginosa *CF isolates. IC14 revealed that the early stage isolates from 1973 had higher expression level of genes involved in type III secretion and exoenzyme activities than other isolates (Figure 4 and Additional file 1, Table S1). More importantly, IC6, IC10 and IC18 revealed adaptations shared by the late stage isolates. IC6 mainly identified antimicrobial peptide resistance related *arn *and *pmr *genes (PA3552-PA3559 and PA4773-PA4782) (Figure 4 and Additional file 1, Table S1). IC10 mainly identified the alginate biosynthesis regulatory *algU *(PA0762), *mucA *(PA0763), *mucB *(PA0764), *mucC *(PA0765) and *algR *(PA5261) genes; the potassium uptake *kdp *genes (PA1632-PA1635) (Figure 4 and Additional file 1, Table S1) and the quorum sensing genes (PA1430-PA1431) (Figure 4 and Additional file 1, Table S1). IC18 mainly identified alginate biosynthesis *alg *genes (PA3540-PA3551) and flagellum and type IV pilus biogenesis genes (Figure 4 and Additional file 1, Table S1).

Besides common adaptations shared by a group of *P. aeruginosa *CF isolates, the ICA also showed that *P. aeruginosa *CF isolates from early infection stage employed multiple patient-specific strategies of adaptation in the CF airways. IC2 revealed that the early stage B12-4 and B12-7 isolates induced the expression of genes related to MexAB-OprM efflux system, iron uptake as well as citronellol/leucine catabolism (Figure 4 and Additional file 1, Table S1). IC4 revealed that the early stage B6-0 and B6-4 isolates up-regulated expression of LPS biosynthesis *wbp *genes (PA3146-PA3159) and down-regulated expression of genes involved in the flagellum biogenesis (Figure 4 and Additional file 1, Table S1). IC16 revealed that the early stage CF114-1973 isolate up-regulated the expression of genes involved in fimbrial biogenesis while down-regulated expression of the PA0632-PA0639 genes (Figure 4 and Additional file 1, Table S1). IC20 revealed that the late stage CF66-2008 isolate up-regulated the expression of the LPS biosynthesis *wbp *genes (PA5448-PA5454) (Figure 4 and Additional file 1, Table S1).

### ICA enhanced identification of co-regulated genes for adaptation of *P. aeruginosa *to the CF airways

**Figure 6:**
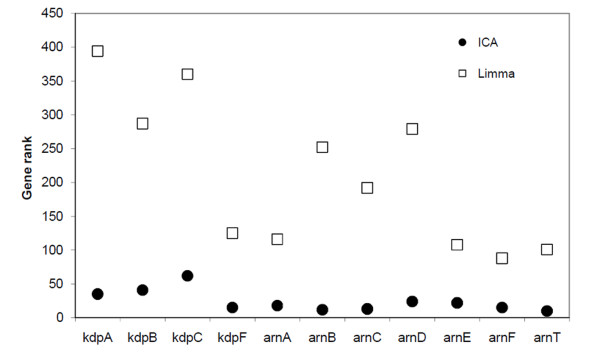
**Enrichment of co-regulated genes with output from ICA and LIMMA analysis**. The ranks of selected genes are plotted.

We further compared the power of ICA and Linear Models for Microarray Data (LIMMA) [16] to identify co-changed genes using the *kdp *genes (PA1632-PA1635) and *arn *genes (PA3552-PA3559) as examples (Figure 6). In ICA analysis, the *kdp *genes and *arn *genes were identified from IC6 and IC10 respectively and they are ranked at the top of the short gene lists generated from these ICs (Figure 6). In contrast, when the *P. aeruginosa *microarray dataset from the early stage isolates and late stage isolates were grouped and compared using LIMMA analysis, the *kdp *genes and *arn *genes are not the most significant genes identified (Figure 6), thus can be easily missed during the analysis. By decomposing and extracting genes from the microarray dataset simultaneously, ICA is superior to established single-gene method LIMMA on identifying novel patterns of co-regulated genes.

## Discussion

Understanding the bacterial adaptation is a great challenge for scientists and medical doctors to battle infectious diseases. Bacterial cells have a high level of mutation rate and can adapt to the dynamic host environments by selecting mutants which are more fit to the condition. Thus, a systematic investigation of the whole gene expression profiles of clinical isolates would be needed for modern diagnostic and treatment of infectious diseases. Fortunately, the rapid progress of DNA sequencing projects has made genome sequences of most of the pathogenic bacteria available now. And this has brought DNA microarray technique as a conventional and high-throughput tool for researchers. However, how to properly and accurately analyze the microarray data and extract useful information is another obstacle for using DNA microarray technique.

In the study here, we have analyzed DNA microarray dataset generated from 26 *P. aeruginosa *strains. ICA was shown to be an efficient approach to identify patient-specific adaptations of *P. aeruginosa *isolates. First of all, ICA decomposes and extracts genes from the microarray dataset simultaneously. Thus, co-regulated genes are more easily identified (Figure 6). Secondly, unlike conventional clustering approaches which group genes based on their expression levels, ICA grouped genes independent of expression levels but in a more biologically meaningful manner.

ICA shows that *P. aeruginosa *clinical isolates employ multiple patient-specific adaption strategies during the early stage infection. Most of these early stage adaptive changes are involved in modification of cell surface molecules and appendages. IC4 reveals that B6-0 and B6-4 isolates enhanced the expression of B-band lipopolysaccharide (LPS) biosynthesis genes while reduced the expression of flagellum biogenesis genes. The B-band LPS is a well known virulence factor which confers *P. aeruginosa *resistance to phagocytosis and serum-mediated killing [17-20]. Loss of flagellum as well as flagellum-mediated motility is documented to render *P. aeruginosa *CF isolates an advantage in the context of immune evasion [21-23]. IC16 reveals that CF114-1973 isolate enhanced the expression of the *cupA *fimbrial gene cluster and the type IV pilus biogenesis cluster. The gene products of these two clusters are required for *P. aeruginosa *adherence and biofilm formation [24-28]. Interestingly, IC16 also reveals the increased expression of *pprB *gene in CF114-1973, which was recently reported as a new regulatory element controlling the *cupE *gene expression and transition between planktonic and community lifestyles in *P. aeruginosa *[29].

ICA facilitates enrichment of co-regulated genes of *P. aeruginosa *CF isolates. For example, IC6 groups the two antimicrobial peptide resistance related gene clusters (*arn *and *pmr*) together. IC18 groups alginate biosynthesis gene cluster PA3540-PA3551 and flagellum biogenesis gene cluster PA1077-PA1086 together. These two gene clusters are impossible to be grouped together by other approaches since they are not localized adjacently in the genome and have different expression levels (one up-regulated and one down-regulated). And this grouping is biologically meaningful since it is well known that alginate regulator inhibits flagellum synthesis gene expression [30-32]. Many genes which encode hypothetical proteins are grouped in IC6, IC10 and IC18. It will be interesting to investigate whether these genes are functionally related with the annotated genes identified in the same ICs.

Since ICA can reveal patient-specific adaptations of *P. aeruginsoa *isolates, it is possible to design patient-specific therapies based on these adaptations. For example, combination of iron chelators and efflux pump inhibitors might be used to inhibit the growth of B12-4 and B12-7, which have high expression levels of genes involved in efflux pump and iron uptake systems [33]. Ligands with high affinity to pili can be used to inhibit adhesion and biofilm formation of the CF114-1973 isolate [34].

## Conclusions

In conclusion, the ICA is shown to be able to extract the most essential features from the complex multiple variant microarray dataset and identify significant genes contribute to these features. Our results show that *P. aeruginosa *employ a diverse set of patient-specific adaption strategies during the early stage infections while certain essential evolutionary events occurred in parallel during the chronic infections in CF infections. The ICA has a great potential in studying large-scale datasets acquired from omics research from different areas.

## Methods

### P. aeruginosa clinical isolates

The *P. aeruginosa *strains were isolated from 6 CF patients with long-term chronic infection and 3 CF patients who were intermittently colonized or recently chronically infected and who were attending the Danish CF Center, Rigshospitalet, Copenhagen. *P. aeruginosa *PAO1 [35] was used as a reference strain.

### DNA microarray

Transcriptomic profiles of clinical isolates were obtained using the Affymetrix *P. aeruginosa *gene chip (Santa Clara, CA) [5,8]. Triplicate experiments were performed for each strain. The microarray raw datasets are accessible at NCBI's Gene Expression Omnibus (GEO) with series accession number GSE31227.

### Mathematical model of gene regulation by ICA

The FastICA package (http://research.ics.tkk.fi/ica/fastica/) was used to analyze the microarray dataset. The microarray gene expression data is considered a linear combination of some independent components which have specific biological interpretations [11]. A *n *× *m *matrix ***X ***is used to represent the microarray gene expression data with *m *gene expressions from *n *clinical isolates. *x_ij _*in *X *is the expression level of the *j-*th gene in the *i-*th isolate. After data have been preprocessed and normalized, the ICA model for gene expression data can be expressed as:

(1) $$\left[\begin{array}{c} x_{1}\left(t\right)\\ \vdots \\ x_{n}\left(t\right) \end{array}\right]=\left[\begin{array}{ccc} a_{11} & \cdots \; & a_{1m}\\ \vdots & \ddots & \vdots \\ a_{n1} & \cdots \; & a_{nm} \end{array}\right]\left[\begin{array}{c} s_{1}\left(t\right)\\ \vdots \\ s_{m}\left(t\right) \end{array}\right]$$

or in matrix notation as:

(2) X=AS

In this ICA model, the columns of ***A ***= [*a_1_, a_2_,..., a_n_*] are the *n *× *n *latent vectors of the gene microarray data. Each column of ***A ***is associated with a specific gene expression mode. ***S ***contains the *n *× *m *gene signatures where the rows of ***S ***are statistically independent to each other. The gene profiles in ***X ***are considered to be a linear mixture of statistically independent components ***S ***combined by an unknown mixing matrix ***A***. Once latent variable matrix ***A ***has been obtained, the corresponding elementary modes can be identified to extract information for classification.

## Competing interests

The authors declare that they have no competing interests.

## Authors' contributions

LY (Lei) carried out the first batch of microarray studies. MR carried out the second batch of microarray studies. LY (Liang) carried out the microarray data analysis and wrote the manuscript. NH provided the strains for the study. SM and LJ participated in the design of the study and helped to draft the manuscript. All authors read and approved the final manuscript.

## Supplementary Material

Additional file 1 **Table S1**. Selected significant genes identified through different latent variables. Click here for file
