# Supplementary material for: Bacterial adaptation during chronic infection revealed by independent component analysis of transcriptomic data
Source: BMC Microbiol. 2011 Aug 18;11:184. doi: 10.1186/1471-2180-11-184 (PMC3224102; doi:10.1186/1471-2180-11-184)
Supplement: Additional file 1 — Table S1. Selected significant genes identified through different latent variables. [file 1471-2180-11-184-S1.DOCX]

Table S1. Selected significant genes identified through different latent variables.

| IC | Locus ID and gene name | Description | Regulation |
| --- | --- | --- | --- |
| 2 | **Antibiotic resistance** |  |  |
| 2 | *PA4596* | probable transcriptional regulator | up |
| 2 | *PA4597 oprJ* | multidrug efflux outer membrane protein OprJ precursor | up |
| 2 | *PA4598 mexD* | RND multidrug efflux transporter MexD | up |
| 2 | *PA4599* *mexC* | RND multidrug efflux membrane fusion protein MexC precursor | up |
| 2 | *PA4600 nfxB* | transcriptional regulator NfxB | up |
| 2 | *PA4601* *morA* | motility regulator | up |
| 2 | **Iron metabolism** |  |  |
| 2  2 | *PA0672* *hemO*  *PA4710* *phuR* | heme oxygenase  haem/haemoglobin uptake receptor PhuR precursor | up  up |
| 2 | *PA2413* *pvdH* | L-2,4-diaminobutyrate:2-ketoglutarate 4-aminotransferase | up |
| 2 | *PA2426 pvdS* | sigma factor PvdS | up |
| 2 | **Citronellol/leucine catabolism** |  |  |
| 2 | *PA2888 atuC* | geranyl-CoA carboxylase, beta-subunit | up |
| 2 | *PA2889* *atuD* | putative citronellyl-CoA dehydrogenase | up |
| 2 | **Others** |  |  |
| 2 | *PA1244* | hypothetical protein | up |
| 2 | *PA2358* | hypothetical protein | up |
| 2 | *PA2411* | probable thioesterase | up |
| 2 | *PA2412* | conserved hypothetical protein | up |
| 2 | *PA2452* | hypothetical protein | up |
| 2 | *Pae_AF241171cds4* | two-component response regulator | up |
|  |  |  |  |
| 4 | **LPS modification** |  |  |
| 4 | *PA3146 wbpK* | probable NAD-dependent epimerase/dehydratase WbpK | up |
| 4 | *PA3147 wbpJ* | probable glycosyl transferase WbpJ | up |
| 4 | *PA3148 wbpI* | UDP-N-acetylglucosamine 2-epimerase WbpI | up |
| 4 | *PA3149 wbpH* | probable glycosyltransferase WbpH | up |
| 4 | *PA3150 wbpG* | LPS biosynthesis protein WbpG | up |
| 4 | *PA3151 hisF2* | imidazoleglycerol-phosphate synthase, cyclase subunit | up |
| 4 | *PA3152 hisH2* | glutamine amidotransferase | up |
| 4 | *PA3155 wbpE* | transaminase, WbpE | up |
| 4 | *PA3159 wbpA* | UDP-N-acetyl-d-glucosamine 6-Dehydrogenase | up |
| 4 | **Flagellum biogenesis** |  |  |
| 4 | *Pae_L81176cds3* | transcription regulator of flagellum assembly | up |
| 4 | *Pae_L81176cds4* | transcription regulator of flagellum assembly | up |
| 4 | *Pae_L81176cds5* | transcription regulator of flagellum assembly | up |
| 4 | *Pae_L81176cds6* | transcription regulator of flagellum assembly | up |
| 4 | *PA1086 flgK* | flagellar hook-associated protein 1 FlgK | down |
| 4 | *PA1087 flgL* | flagellar hook-associated protein type 3 FlgL | down |
| 4 | *PA1088* | hypothetical protein | down |
| 4 | *PA1089* | conserved hypothetical protein | down |
| 4 | *PA1090* | hypothetical protein | down |
| 4 | *PA1091* *fgtA* | flagellar glycosyl transferase, FgtA | down |
| 4 | *PA1092 fliC* | flagellin type B | down |
| 4 | *PA1093* | hypothetical protein | down |
| 4 | *PA1094 fliD* | flagellar capping protein FliD | down |
| 4 | *PA1095* | hypothetical protein | down |
| 4 | *PA1096* | hypothetical protein | down |
| 4 | **Others** |  |  |
| 4 | *PA0642* | hypothetical protein | up |
| 4 | *PA0646* | hypothetical protein | up |
| 4 | *PA0983* | conserved hypothetical protein | up |
| 4 | *PA1372* | hypothetical protein | up |
| 4 | *Pae_AF241171cds21* | two-component signal transduction | up |
| 4 | *Pae_M21651cds* | cell adhesion | up |
| 4 | *Pae_M57501cds_g* | unkrown function | up |
| 4 | *ig_3545880_3545073* | unkrown function | up |
| 4 | *Pae_orfA_vioA* | unkrown function | up |
| 4 | *Pae_orfB* | unkrown function | up |
| 4 | *Pae_orfC* | unkrown function | up |
| 4 | *Pae_orfD* | unkrown function | up |
| 4 | *Pae_orfE* | unkrown function | up |
| 4 | *Pae_orfF* | unkrown function | up |
| 4 | *Pae_orfG* | unkrown function | up |
| 4 | *Pae_orfH* | unkrown function | up |
| 4 | *Pae_orfJ* | unkrown function | up |
| 4 | *Pae_orfK* | unkrown function | up |
| 4 | *Pae_flgL* | unkrown function | up |
| 4 | *Pae_orfM* | unkrown function | up |
| 4 | *Pae_orfI* | unkrown function | up |
|  |  |  |  |
| 16 | **Fimbrial biogenesis** |  |  |
| 16 | *PA2126* | conserved hypothetical protein | up |
| 16 | *PA2127* | conserved hypothetical protein | up |
| 16 | *PA2128 cupA1* | fimbrial subunit CupA1 | up |
| 16 | *PA2129 cupA2* | chaperone CupA2 | up |
| 16 | *PA2130 cupA3* | usher CupA3 | up |
| 16 | *PA2131 cupA4* | fimbrial subunit CupA4 | up |
| 16 | *PA2132 cupA5* | chaperone CupA5 | up |
| 16 | *PA4550 fimU* | type 4 fimbrial biogenesis protein FimU | up |
| 16 | *PA4551 pilV* | type 4 fimbrial biogenesis protein PilV | up |
| 16 | *PA4552 pilW* | type 4 fimbrial biogenesis protein PilW | up |
| 16 | *PA4554 pilY1* | type 4 fimbrial biogenesis protein PilY1 | up |
| 16 | *PA4555 pilY2* | type 4 fimbrial biogenesis protein PilY2 | up |
| 16  16 | *PA4556 pilE*  *PA4296 pprB* | type 4 fimbrial biogenesis protein PilE  two-component response regulator, PprB | up  up |
| 16 | **Others** |  |  |
| 16 | *PA2223* | hypothetical protein | up |
| 16 | *PA2224* | hypothetical protein | up |
| 16 | *PA2225* | hypothetical protein | up |
| 16 | *PA2226* | hypothetical protein | up |
| 16 | *PA2227 vqsM* | AraC-type transcriptional regulator VqsM | up |
| 16 | *PA2228* | hypothetical protein | up |
| 16 | *PA0632* | hypothetical protein | down |
| 16 | *PA0633* | hypothetical protein | down |
| 16 | *PA0634* | hypothetical protein | down |
| 16 | *PA0635* | hypothetical protein | down |
| 16 | *PA0636* | hypothetical protein | down |
| 16 | *PA0637* | conserved hypothetical protein | down |
| 16 | *PA0638* | conserved hypothetical protein | down |
| 16 | *PA0639* | conserved hypothetical protein | down |
| 16 | *PA2877* | probable transcriptional regulator | up |
| 16 | *PA4141* | hypothetical protein | up |
| 16 | *PA5167* | probable c4-dicarboxylate-binding protein | up |
| 16 | *Pae_U97065cds3* | lipid metabolic process | up |
| 16 | *ig_1063544_1064555* | unkrown function | up |
|  |  |  |  |
| 20 | **LPS modification** |  |  |
| 20 | *PA5448 wbpY* | glycosyltransferase WbpY | up |
| 20 | *PA5449 wbpX* | glycosyltransferase WbpX | up |
| 20 | *PA5450 wzt* | ABC subunit of A-band LPS efflux transporter | up |
| 20 | *PA5451 wzm* | membrane subunit of A-band LPS efflux transporter | up |
| 20 | *PA5452 wbpW* | phosphomannose isomerase/GDP-mannose WbpW | up |
| 20 | *PA5453 gmd* | GDP-mannose 4,6-dehydratase | up |
| 20 | *PA5454 rmd* | oxidoreductase Rmd | up |
| 20 | **Others** |  | up |
| 20 | *PA2317* | probable oxidoreductase | up |
| 20 | *PA2318* | hypothetical protein | up |
| 20 | *PA3013 foaB* | fatty-acid oxidation complex beta-subunit | up |
| 20 | *PA3014 faoA* | fatty-acid oxidation complex alpha-subunit | up |
| 20 | *PA3569 mmsB* | 3-hydroxyisobutyrate dehydrogenase | up |
| 20 | *PA3570 mmsA* | methylmalonate-semialdehyde dehydrogenase | up |
| 20 | *PA3922* | conserved hypothetical protein | up |
| 20 | *PA3923* | hypothetical protein | up |
| 20 | *PA4071* | hypothetical protein | up |
| 20 | *PA4072* | probable amino acid permease | up |
| 20 | *PA4073* | probable aldehyde dehydrogenase | up |
| 20 | *PA4794* | hypothetical protein | up |
| 20 | *PA4795* | hypothetical protein | up |
| 20 | *PA4796* | hypothetical protein | up |
| 20 | *PA0506* | probable acyl-CoA dehydrogenase | down |
| 20 | *PA0507* | probable acyl-CoA dehydrogenase | down |
| 20 | *PA0508* | probable acyl-CoA dehydrogenase | down |
| 20 | *PA5506* | hypothetical protein | down |
| 20 | *PA5507* | hypothetical protein | down |
| 20 | *PA5508* | probable glutamine synthetase | down |
| 20 | *PA5509* | hypothetical protein | down |
| 20 | *PA0782 putA* | proline dehydrogenase PutA | down |
| 20 | *PA1070 braG* | branched-chain amino acid transport protein BraG | up |
| 20 | *PA4100* | probable dehydrogenase | up |
|  |  |  |  |
| 22 | *PA4834* | hypothetical protein | up |
| 22 | *PA4835* | hypothetical protein | up |
| 22 | *PA4836* | hypothetical protein | up |
| 22 | *PA4837* | probable outer membrane protein precursor | up |
| 22 | *PA4838* | hypothetical protein | up |
| 22 | *PA0747* | probable aldehyde dehydrogenase | up |
| 22 | *PA1281 cobV* | cobalamin (5'-phosphate) synthase | up |
| 22 | *PA1340* | amino acid ABC transporter membrane protein | up |
| 22 | *PA1759* | probable transcriptional regulator | up |
| 22 | *PA1985 pqqA* | pyrroloquinoline quinone biosynthesis protein A | up |
| 22 | *PA1990* | probable peptidase | up |
| 22 | *PA2327* | probable permease of ABC transporter | up |
| 22 | *PA3222* | hypothetical protein | up |
| 22 | *PA3234* | probable sodium:solute symporter | up |
| 22 | *PA3235* | conserved hypothetical protein | up |
| 22 | *PA3780* | hypothetical protein | up |
| 22 | *PA4220* | hypothetical protein | up |
| 22 | *PA4908* | hypothetical protein | up |
| 22 | *PA5384* | probable lipolytic enzyme | up |
| 22 | *ig_3874653_3873835_r* | unkrown function | up |
|  |  |  |  |
| 14 | **Type III secretion** |  |  |
| 14 | *PA1690 pscU* | translocation protein in type III secretion | up |
| 14 | *PA1691 pscT* | translocation protein in type III secretion | up |
| 14 | *PA1692* | probable translocation protein in type III secretion | up |
| 14 | *PA1693 pscR* | translocation protein in type III secretion | up |
| 14 | *PA1694 pscQ* | translocation protein in type III secretion | up |
| 14 | *PA1695 pscP* | translocation protein in type III secretion | up |
| 14 | *PA1696 pscO* | translocation protein in type III secretion | up |
| 14 | *PA1697* | ATP synthase in type III secretion system | up |
| 14 | *PA1698 popN* | ATP synthase in type III secretion system | up |
| 14 | *PA1699* | conserved hypothetical protein in type III secretion | up |
| 14 | *PA1700* | conserved hypothetical protein in type III secretion | up |
| 14 | *PA1701* | conserved hypothetical protein in type III secretion | up |
| 14 | *PA1702* | conserved hypothetical protein in type III secretion | up |
| 14 | *PA1703 pcrD* | type III secretory apparatus protein PcrD | up |
| 14 | *PA1704 pcrR* | transcriptional regulator protein PcrR | up |
| 14 | *PA1705 pcrG* | regulator in type III secretion | up |
| 14 | *PA1706 pcrV* | type III secretion protein PcrV | up |
| 14 | *PA1707 pcrH* | regulatory protein PcrH | up |
| 14 | *PA1708 popB* | translocator protein PopB | up |
| 14 | *PA1709 popD* | Translocator outer membrane protein PopD precursor | up |
| 14 | *PA1710 exsC* | anti-antiactivator and type III secretion chaperone | up |
| 14 | *PA1711 exsE* | negative regulator of type III secretion gene | up |
| 14 | *PA1712 exsB* | exoenzyme S synthesis protein B | up |
| 14 | *PA1713 exsA* | primary transcriptional regulator of type III secretion genes | up |
| 14 | *PA1714 exsD* | antiactivator that binds to ExsA | up |
| 14 | *PA1715 pscB* | type III export apparatus protein | up |
| 14 | *PA1716 pscC* | Type III secretion outer membrane protein PscC precursor | up |
| 14 | *PA1717 pscD* | type III export protein PscD | up |
| 14 | *PA1718 pscE* | type III export protein PscE | up |
| 14 | *PA1719 pscF* | type III export protein PscF | up |
| 14 | *PA1720 pscG* | type III export protein PscG | up |
| 14  14  14  14  14 | *PA1721 pscH* | type III export protein PscH | up |
|  | *PA1722 pscI* | type III export protein PscI | up |
|  | *PA1723 pscJ* | type III export protein PscJ | up |
|  | *PA1724 pscK* | type III export protein PscK | up |
|  | *PA1725 pscL* | type III export protein PscL | up |
| 14 | *PA0044 exoT* | exoenzyme T | up |
| 14 | *PA2191 exoY* | adenylate cyclase ExoY | up |
| 14 | *PA3841 exoS* | exoenzyme S | up |
| 14 | *PA3842* | probable chaperone | up |
| 6 | **Antimicrobial peptide tolerance** |  |  |
| 6 | *PA3552 arnB* | antimicrobial peptide resistant | up |
| 6 | *PA3553 arnC* | antimicrobial peptide resistant | up |
| 6 | *PA3554 arnA* | antimicrobial peptide resistant | up |
| 6 | *PA3555 arnD* | antimicrobial peptide resistant | up |
| 6 | *PA3556 arnT* | inner membrane L-Ara4N transferase ArnT (or pmrK) | up |
| 6 | *PA3557 arnE* | antimicrobial peptide resistant | up |
| 6 | *PA3558 arnF* | antimicrobial peptide resistant | up |
| 6 | *PA3559* | probable nucleotide sugar dehydrogenase | up |
| 6 | *PA4773* | hypothetical protein | up |
| 6 | *PA4774* | hypothetical protein | up |
| 6 | *PA4775* | hypothetical protein | up |
| 6 | *PA4776 pmrA* | two-component regulator system response regulator PmrA | up |
| 6 | *PA4777 pmrB* | two-component regulator system signal sensor kinase PmrB | up |
| 6 | *PA4781* | cyclic di-GMP phosphodiesterase | up |
| 6 | *PA4782* | hypothetical protein | up |
| 6 | **Others** |  |  |
| 6 | *PA0909* | hypothetical protein | up |
| 6 | *PA0910* | hypothetical protein | up |
| 6 | *PA0911* | hypothetical protein | up |
| 6 | *PA1559* | hypothetical protein | up |
| 6 | *PA1560* | hypothetical protein | up |
| 6 | *PA2317* | probable oxidoreductase | up |
| 6 | *PA2318* | hypothetical protein | up |
| 6 | *PA4072* | probable amino acid permease | up |
| 6 | *PA4073* | probable aldehyde dehydrogenase | up |
| 6 | *PA4357* | conserved hypothetical protein | up |
| 6 | *PA4358* | probable ferrous iron transport protein | up |
| 6 | *PA4359* | conserved hypothetical protein | up |
| 6 | *PA0640* | probable bacteriophage protein | up |
| 6 | *PA3205* | hypothetical protein | up |
| 6 | *PA3602* | conserved hypothetical protein | up |
| 6 | *PA4100* | probable dehydrogenase | up |
|  |  |  |  |
| 10 | **Alginate biosynthesis** |  | up |
| 10 | *PA0762 algU* | sigma factor AlgU | up |
| 10 | *PA0763 mucA* | anti-sigma factor MucA | up |
| 10 | *PA0764 mucB* | negative regulator for alginate biosynthesis MucB | up |
| 10 | *PA0765 mucC* | positive regulator for alginate biosynthesis MucC | up |
| 10 | *PA5261 algR* | alginate biosynthesis regulatory protein AlgR | up |
| 10 | **Potassium uptake** |  |  |
| 10 | *PA1632 kdpF* | prokaryotic K^+^-transporting system | up |
| 10 | *PA1633 kdpA* | prokaryotic K^+^-transporting system | up |
| 10 | *PA1634 kdpB* | prokaryotic K^+^-transporting system | up |
| 10 | *PA1635 kdpC* | prokaryotic K^+^-transporting system | up |
| 10 | **Quorum sensing** |  |  |
| 10 | *PA1430 lasR* | transcriptional regulator LasR | down |
| 10 | *PA1432 lasI* | autoinducer synthesis protein LasI | down |
| 10 | *PA1431 rsaL* | quorum-sensing negative regulator RsaL | down |
| 10 | **Others** |  |  |
| 10 | *PA4101 bfmR* | two-component regulatory systems | up |
| 10 | *PA4102 bfmS* | two-component regulatory systems | up |
| 10 | *PA4103* | hypothetical protein | up |
| 10 | *PA4104* | conserved hypothetical protein | up |
| 10 | *PA4105* | hypothetical protein | up |
| 10 | *PA4106* | conserved hypothetical protein | up |
| 10 | *PA4107* | hypothetical protein | up |
| 10 | *PA4110 ampC* | beta-lactamase precursor | up |
| 10 | *PA0062* | hypothetical protein | up |
| 10 | *PA0833* | hypothetical protein | up |
| 10 | *PA1323* | hypothetical protein | up |
| 10 | *PA1324* | hypothetical protein | up |
| 10 | *PA1471* | hypothetical protein | up |
| 10 | *PA1592* | hypothetical protein | up |
| 10 | *PA2317* | probable oxidoreductase | up |
| 10 | *PA2318* | hypothetical protein | up |
| 10 | *PA2562* | hypothetical protein | up |
| 10 | *PA2779* | hypothetical protein | up |
| 10 | *PA3691* | hypothetical protein | up |
| 10 | *PA3692* | Lipotoxon F | up |
| 10 | *PA3819* | conserved hypothetical protein | up |
| 10 | *PA4876 osmE* | osmotically inducible lipoprotein OsmE | up |
| 10 | *PA4972* | hypothetical protein | up |
| 10 | *PA5212* | hypothetical protein | up |
|  |  |  |  |
| 18 | **Alginate biosynthesis** |  |  |
| 18 | *PA3540 algD* | GDP-mannose 6-dehydrogenase AlgD | up |
| 18 | *PA3541* | alginate biosynthesis protein Alg8 | up |
| 18 | *PA3542* | alginate biosynthesis protein Alg44 | up |
| 18 | *PA3543 algK* | alginate biosynthetic protein AlgK precursor | up |
| 18 | *PA3544 algE* | alginate production outer membrane protein AlgE precursor | up |
| 18 | *PA3545 algG* | alginate-c5-mannuronan-epimerase AlgG | up |
| 18 | *PA3546 algX* | alginate biosynthesis protein AlgX | up |
| 18 | *PA3547 algL* | poly(beta-d-mannuronate) lyase precursor AlgL | up |
| 18 | *PA3548 algI* | alginate o-acetyltransferase AlgI | up |
| 18 | *PA3549 algJ* | alginate o-acetyltransferase AlgJ | up |
| 18 | *PA3550 algF* | alginate o-acetyltransferase AlgF | up |
| 18 | *PA3551 algA* | guanosine 5'-diphospho-D-mannose pyrophosphorylase | up |
| 18 | **Motilities** |  |  |
| 18 | *PA1077 flgB* | flagellar basal-body rod protein FlgB | down |
| 18 | *PA1078 flgC* | flagellar basal-body rod protein FlgC | down |
| 18 | *PA1079 flgD* | flagellar basal-body rod modification protein FlgD | down |
| 18 | *PA1080 flgE* | flagellar hook protein FlgE | down |
| 18 | *PA1081 flgF* | flagellar basal-body rod protein FlgF | down |
| 18 | *PA1082 flgG* | flagellar basal-body rod protein FlgG | down |
| 18 | *PA1084 flgI* | flagellar P-ring protein precursor FlgI | down |
| 18 | *PA1086 flgK* | flagellar hook-associated protein 1 FlgK | down |
| 18 | *PA1092 fliC* | flagellin type B | down |
| 18 | *PA1094 fliD* | flagellar capping protein FliD | down |
| 18 | *PA1098 fleS* | two-component sensor | down |
| 18 | *PA1099 fleR* | two-component response regulator | down |
| 18 | *PA1100 fliE* | flagellar hook-basal body complex protein FliE | down |
| 18 | *PA1101 fliF* | flagella M-ring outer membrane protein precursor | down |
| 18 | *PA5044 pilM* | type 4 fimbrial biogenesis protein PilM | down |
| 18 | **Others** |  |  |
| 18 | *PA0062* | hypothetical protein | up |
| 18 | *PA1784* | hypothetical protein | up |
| 18 | *PA1985 pqqA* | pyrroloquinoline quinone biosynthesis protein A | up |
| 18 | *PA2412* | conserved hypothetical protein | up |
| 18 | *PA2485* | hypothetical protein | up |
| 18 | *PA2486* | hypothetical protein | up |
| 18 | *PA3049 rmf* | ribosome modulation factor | up |
| 18 | *PA4154* | conserved hypothetical protein | up |
| 18 | *PA5182* | hypothetical protein | up |
| 18 | *PA5183* | hypothetical protein | up |
| 18 | *PA5212* | hypothetical protein | up |
| 18 | *PA1132* | hypothetical protein | down |
| 18 | *PA3662* | hypothetical protein | down |
| 18 | *PA3905* | hypothetical protein | down |
| 18 | *PA3906* | hypothetical protein | down |
| 18 | *PA4310 pctB* | chemotactic transducer PctB | down |
